# Current status and advances in zinc anodes for rechargeable aqueous zinc-air batteries

**DOI:** 10.1080/14686996.2024.2448418

**Published:** 2025-01-31

**Authors:** Muhammad Afiq Irfan Mohd Shumiri, Abdillah Sani Mohd Najib, Nor Akmal Fadil

**Affiliations:** a Materials Research and Consultancy Group, Faculty of Mechanical Engineering, Universiti Teknologi Malaysia, Johor Bahru, Malaysia; b Department of Materials, Manufacturing and Industrial Engineering, Faculty of Mechanical Engineering, Universiti Teknologi Malaysia, Johor Bahru, Malaysia

**Keywords:** Zinc-air batteries, zinc anode, dendrite-free, surface modification, structural design, cycle stability

## Abstract

To promote sustainable development and reduce fossil fuel consumption, there is a growing demand for high-performance, cost-effective, safe and environmentally friendly batteries for large-scale energy storage systems. Among the emerging technologies, zinc-air batteries (ZABs) have attracted significant interest. By integrating the principles of traditional zinc-ion batteries and fuel cells, ZABs offer remarkably high theoretical energy density at lower production cost compared to the current state-of-the-art lithium-ion batteries (LIBs). However, the critical challenge remains in developing high-performance zinc anode. Herein, this review provides a comprehensive analysis of the current status and advancements in zinc anodes for rechargeable aqueous ZABs. We begin by highlighting the major challenges and underlying mechanisms associated with zinc anodes including issues such as uneven zinc deposition, dendrite growth and hydrogen evolution reaction. Then, this review discusses the recent advancements in zinc anode modifications, focusing on strategies such as alloying, surface porosity and zincophilicity. By reviewing the latest research, we also identify existing gaps and pose critical questions that need further exploration to push the field forward. The goal of this review is to inspire new research directions and promote the development of more efficient zinc anodes.

## IMPACT STATEMENT

This review helps to inspire new approaches and encourage further research in developing more efficient zinc anodes for zinc-air batteries, paving the way for better energy storage solutions.

## GRAPHICAL ABSTRACT

**Figure:**
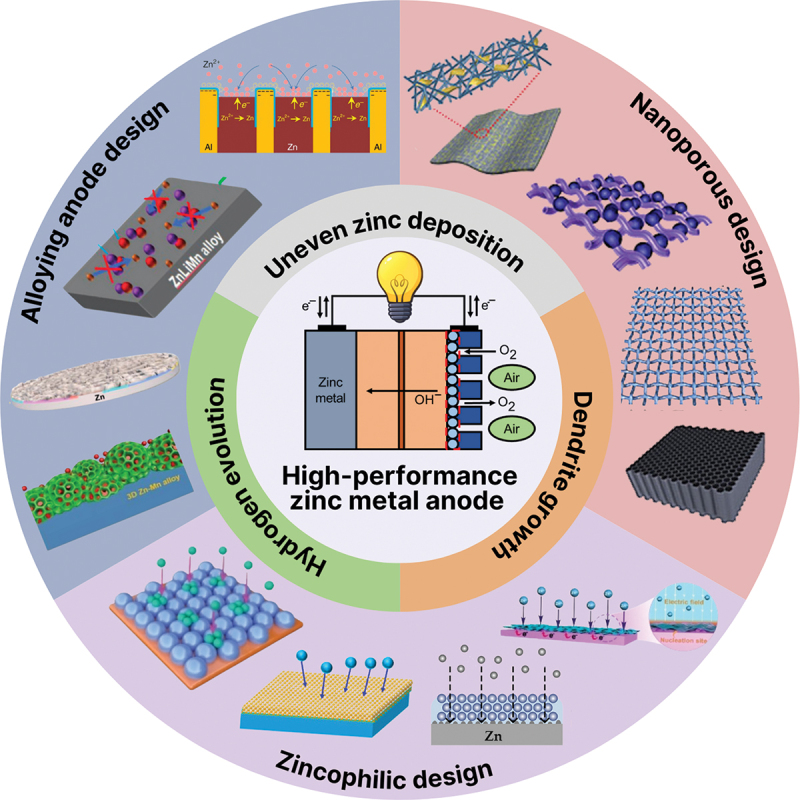

## Introduction

**Figure 1.**
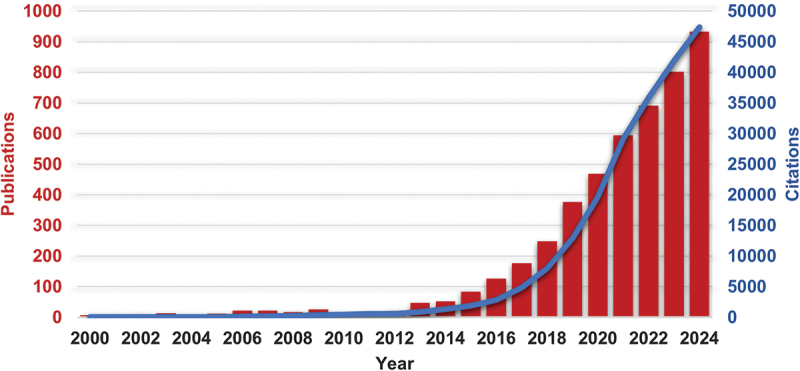
The number of publications and citations over the past decade related to the keyword ‘zinc-air batteries’ from all databases search in Web of Science platform.

In the global effort to reduce fossil fuels consumption, the demand for high performance, inexpensive, safe and environmentally friendly batteries for energy storage system is growing. Currently, state-of-the-art lithium-ion batteries (LIBs) are likely to maintain the market dominance for at least the next decade due to their benefits in terms of energy capacity and cyclability [1]. However, the current state of LIBs has almost reached its limits, raising the question of further advancement [2]. Indeed, LIBs remain costly and the potential future shortage of lithium used in the production emphasizes the necessity for developing batteries with improved cost-effectiveness and material availability [3]. Additionally, LIBs pose significant safety risk as it can catch fire and explode during operation. These challenges highlight the critical need for research and development efforts toward alternative battery technology which is zinc-air battery (ZABs). In the ongoing research, ZABs have received great interest. The field of study on ZABs continue to grow progressively as a potential energy storage system. It can be seen from Figure 1 that the publications and citations related to ZABs show significant increase from 2013 until now.

Zn is the only alternative metal among Li, Al, Fe, Mg, K and Na that can be used directly as the anode because it can undergo stable plating and stripping processes in aqueous electrolytes [4]. Anodes made of Li, Mg, K and Na are incompatible in aqueous systems because these metals react violently in water [5]. Consequently, the reactive activity progressively increases and leads to the irreversible consumption of electrode materials and electrolyte [6]. As a result, aqueous ZABs are considered a promising option for future energy storage system. Although ZABs may not offer the highest theoretical energy density among metal-air batteries, the technical feasibility makes them practical for real-world application [7]. At this point, ZABs are widely used in both everyday life and research. For example, it has been commercialized in small-scale applications like button and coin cells for hearing aids application. Other than that, the company Fluidic Energy has pioneered the commercialization of rechargeable ZABs, successfully deploying them in critical backup power applications. Notably, the cost of ZABs present a significant advantage, being only 1/4 of lead-acid batteries and even 1/17 of LIBs due to the abundant availability of zinc as the raw material. Additionally, the discharge process is stable which results in consistent output voltage. ZABs are considered safe and environmentally friendly with alkaline aqueous electrolytes such as KOH and NaOH.

Initially, academic research focused primarily on cathode materials, with most efforts directed towards advancing the material and structural design of bifunctional oxygen catalysts of the gas diffusion electrode [8]. However, the critical challenge remains in achieving high-performance zinc anode. There is insufficient research into anode modification to enhance the electrochemical performance and cycling stability. The mass of zinc anode is far greater than needed to balance the capacity of cathode [4]. Moreover, the active material of zinc on the anode surface is directly participate in redox reaction during charge and discharge process. Consequently, modification on zinc anode is important to enhance the overall performance of aqueous ZABs. The performance of zinc anode has been improved in recent years by using various innovative methods and materials. Conducting a comprehensive discussion of these strategies and the connection between material structure can lead to more efficient material design and optimization. Such a review would also provide an up-to-date summary of the latest developments in this significant area of research.

This review provides a comprehensive discussion on the current status and advancements in zinc anode for rechargeable aqueous ZABs. It begins by addressing the key challenges and underlying mechanisms associated with zinc anodes that focuses on issues such as uneven zinc deposition, dendrite growth and hydrogen evolution reaction. These challenges are critical as they directly impact the performance and longevity of ZABs. Finally, this review summarizes three current advancements in zinc anode modifications including alloying the anode, surface porosity and zincophilicity. Each of these modifications aims to tackle the identified challenges and improve the overall performance of zinc anodes. By evaluating recent literature, this review identifies gaps and poses research questions that need to be addressed to further advance the field. The goal of this review is to present new approaches that will encourage further research in developing more efficient zinc anodes for aqueous ZABs.

## Key challenges for zinc anode

**Figure 2.**
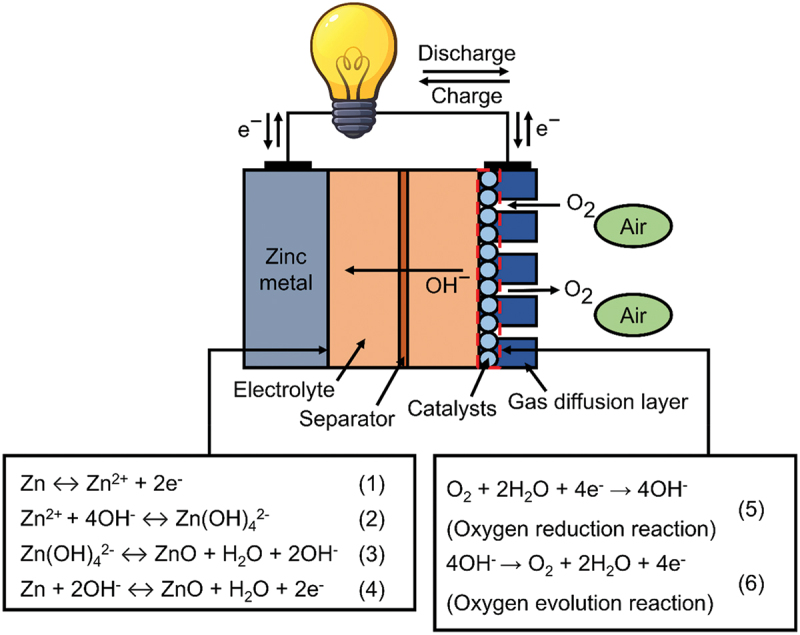
Structure of the rechargeable alkaline aqueous zinc-air battery with reaction mechanisms at the zinc metal anode and air cathode.

Zinc remains the most suitable anode due to chemical stability in water compared to other metal. The stripping and plating reactions of zinc enable ZABs to achieve larger capacity and higher energy density relative to monovalent charge carriers. Figure 2 illustrates the structure and reaction equation of rechargeable ZABs. In anode reaction, zinc initially releases two electrons during discharge process to generate Zn^2+^ as shown by Equation [1]. Then, Zn^2+^ is further combined with OH^−^ to form soluble zincate ions (Zn(OH)_4_^2-^) following Equation [2]. As the concentration of zincate ions become supersaturated on the anode surface, it undergoes decomposition into H_2_O, OH^−^ and insoluble zinc oxide (ZnO) (Equation [3]) [9]. Equation [4] represents overall anode reaction with equilibrium potential of −1.25 V vs. reversible hydrogen electrode (RHE) based on the redox pair Zn/Zn^2 +^. The electrons released from anode move through the external circuit when a load is connected to the battery. At the same time, oxygen reduction reaction (ORR) occurs at the air cathode as it accepts the electrons from current collector (Equation 5). The production of OH^−^ spontaneously move from cathode to anode surface driven by concentration polarization [10]. Therefore, it is necessary to use electrolyte with excellent ionic conductivity to facilitate these electrochemical processes. During charging process, the reactions are reversed and oxygen evolution reaction (OER) occurs at the air cathode (Equation 6).

The theoretical energy density of ZABs is high, significantly surpassing that of LIBs with gravimetric and volumetric energy density of 1218 Wh/kg and 6136 Wh/L, respectively [11]. Zinc is inexpensive and abundant metal, widely available in the earth’s crust and extensively processed by the industry [12,13]. Metallic zinc has excellent electrical conductivity with a resistivity of 5.90 × 10^− 8^ Ω/m. This high conductivity enhances electron transport and promotes the efficient charge-discharge process. Additionally, zinc is non-toxic, highly stable, poses no risk of fire and environmentally friendly which makes the production and utilization of ZABs safe and sustainable [14,15]. However, there are several issues encountered during operation in aqueous electrolyte. The challenges on anode including uneven zinc deposition, dendrite growth and hydrogen evolution reaction are the most critical across the entire field of zinc-based energy storage technologies. Owing to the fact that ZABs rely on similar electrochemical processes on the anode side, the challenges are generalized beyond ZABs due to the unique design and operational characteristics.

### Uneven zinc deposition

ZABs achieve optimal performance in highly alkaline environments, as the air cathode exhibits superior activity and stability for both ORR and OER [16]. Advanced electrocatalyst materials have demonstrated excellent electrochemical performance in alkaline media, characterized by low overpotential and high peak power density [17]. While ZABs are capable of operating in neutral electrolytes, their performance is compromised by slower reaction kinetics, resulting in lower power density and reduced stability of the air cathode [18]. Acidic environments are even less favourable as the dissolution of metal catalysts leads to rapid air cathode degradation and significantly shortened cycle life [19]. For this reason, alkaline electrolytes are the most suitable for ZABs, offering enhanced catalytic performance and durability. However, on the anode side, it is necessary to regulate the mass transfer and ion diffusion due to the fact that zinc has high solubility in strong alkaline electrolyte [20]. Deposition of zinc involves four different steps which are mass transport, desolvation, nucleation and crystal growth [19]. During electrodeposition, molecular diffusion allows the transfer of zincate ions and produce dense concentrated boundary layer around the anode [21]. This condition promotes uneven deposition of zinc due to polarization on the anode surface. During charging process, concentration gradient will occur due to different ionic concentration between the anode surface and bulk electrolyte [22].

According to Nernst – Planck equation, the concentration gradient, electric field and convection intensity are parameters that influence uneven zinc deposition. These parameters collectively contribute to the force of ion transfer which is diffusion flux in the electrolyte as described by following equation.

(1) $$J=\frac{\mathrm{qCD}}{\mathrm{kT}}\frac{\mathrm{dv}}{\mathrm{dx}}-D\frac{\mathrm{dC}}{\mathrm{dx}}+CV_{x}$$

where J is the diffusion flux, q is the unit charge, C is the concentration, D is the coefficient of diffusion, k is the Boltzmann constant, T is the temperature, V is the electric potential, x is the distance and V_x_ is the convective velocity [23]. Ions are forced to move more rapidly by external electric field and nucleate on the anode [22]. High current density induces uneven nucleation process and results in irregularity in charge transportation [24]. This irregularity can lead to non-uniform distribution of ion flux and various nucleation barrier sites. Under conditions of unrestricted ion diffusion, zinc ions tend to preferentially adsorb and concentrate on the higher active sites.

**Figure 3.**
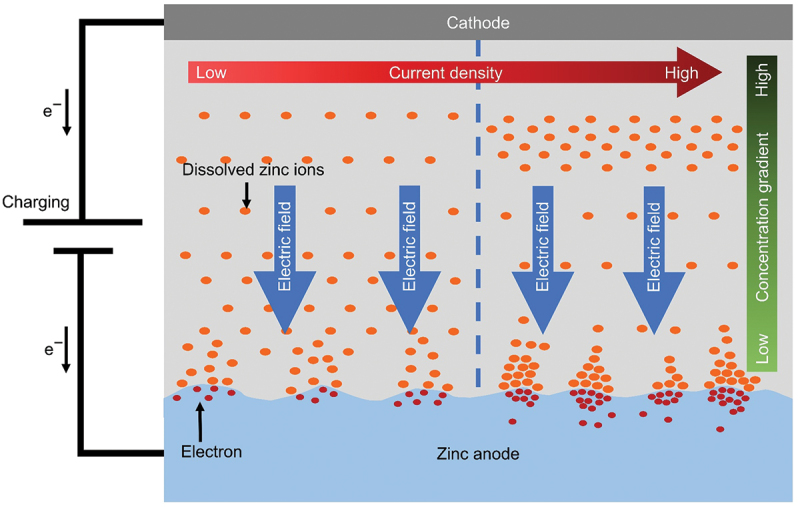
Schematic illustration of zinc nucleation process during electrodeposition under the influence of electric field and current density.

Zinc ions close to the anode surface initially move and adsorb to the nucleation sites under the influence of electric field force. When overpotential is higher than the energy barrier of nucleation, zinc ions receive electrons and deposit on the nucleation sites. High surface energy of nucleation sites helps the accumulation of zinc ions which promote the formation of protrusions [7]. At the tip of protrusions, the accumulation of charge causes large overpotential which makes it easier for zinc ions to deposit compared to a smooth surface. This nucleation process is described in Figure 3. The nucleation and growth process of zinc significantly influence the quality and granularity of electrodeposition [25]. Faster nucleation rates and finer crystal grains of deposition are produced by larger overpotentials. On the other hand, coarser grains are produced by smaller overpotentials. Desolvation of zinc ions is another important factor as the changes in coordination shell of electrolyte components can influence this process. Unbalanced external factors such as gravity and electric field can unevenly distribute active species and increase nucleation in specific locations [26]. Similarly, the rate and quality of crystal growth are influenced by convection intensity and gravitational effects within the electrolyte [23]. In situation of low convection, the concentration of zinc ions may fluctuate significantly and result in concentration polarization.

In ZAB systems, uneven zinc deposition at the anode side introduces localized variations in OH^−^ concentration within the electrolyte. This condition creates significant electrochemical and transport-related challenges that directly affect the performance of air cathode. Specifically, non-uniform zinc deposition results in regions of OH^−^ accumulation near the catalyst [27,28]. These gradients induce uneven ionic fluxes through the electrolyte, leading to inconsistent ionic supply in ZAB systems [29]. The imbalance can adversely affect ORR kinetic, reducing the efficiency of bifunctional catalytic performance [27]. ORR is highly sensitive to the local availability of OH^−^ ions. The mechanism is complex because it incorporates with multiple electrons in the elementary reaction and involve different intermediates. Oxygen molecules adsorbed on the catalyst surface are supposed to undergo reduction to OH^−^ through 4-electron pathway (O_2_ +2 H_2_O +4e^−^ → 4OH^−^). However, due to uneven ionic flux, the system may shift towards the less efficient 2-electron pathway (O_2_ + H_2_O +2e^−^ → HO_2_^−^ + OH^−^, HO_2_^−^ + H_2_O +2e^−^ → 3OH^−^) [30]. This transition results in significant energy loss and reduced discharge durability of ZABs [31].

However, several methods have been proposed to prevent nonuniform zinc deposition based on the diffusion-controlled mechanism. Researchers have employed pulse charging and flowing electrolytes to decrease zincate concentration gradient. A study found the distribution of ions near the anode can be adjusted by pulse charging by introducing a short rest time to restore the zinc concentration [32]. Similarly, flowing electrolyte helps to promote the migration of zinc ions and optimize the distribution on the anode surface [33]. Changing ion mass transfer from diffusion to convection by flowing electrolyte can eliminate non-uniform zinc deposition and bring the system closer to equilibrium [21]. During charging process, the electrolyte containing higher concentration of zincate ions flow in to manipulate the concentration gradient and result in uniform zinc deposition. The electrolyte is pumped and circulated through an external system of pipes and pumps. However, sustaining the power for electrolyte circulation requires an external pumping system and electric energy. This presents a challenge when applied to large scale grid energy storage systems with limited space and weight requirements [34].

### Dendrite growth

**Figure 4.**
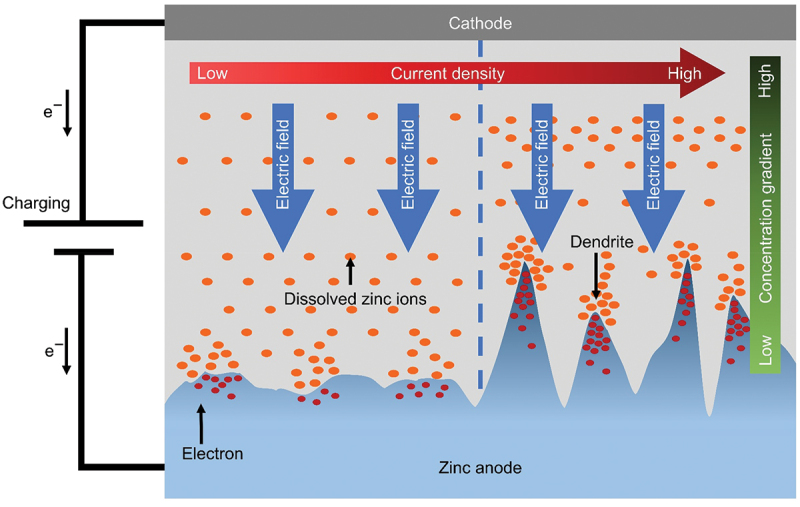
Schematic illustration of zinc dendrite growth due to uneven deposition during charging process.

The formation of dendrites poses significant challenge for rechargeable ZABs. During charging process, solid ZnO is initially reduced to soluble zincate ions (Zn(OH)_4_^2-^). After that, this soluble compound undergoes further reduction to form zinc metal on the surface of anode. However, due to unequal distribution of current and potential on the zinc surface, as well as gravity effect, the soluble zincate ions tend to disperse randomly [21]. Uneven deposition of zinc is inevitable under the influence of electric field and natural gravitational field [35]. This result in changes to the shape of anode and produce rough anode surface as shown in Figure 4. Dendritic growth at the electrode has a certain memory effect [26]. As charging progresses, dendrites are constantly formed because the soluble zincate ions preferentially adsorbed and reduced on the small dendritic tip with sharp end structure. Zn atomic clusters would develop on these locations and promote the formation of dendrites because of the tip effect [36]. Therefore, the distribution of dendrites would promote the subsequent dendrite growth.

**Figure 5.**
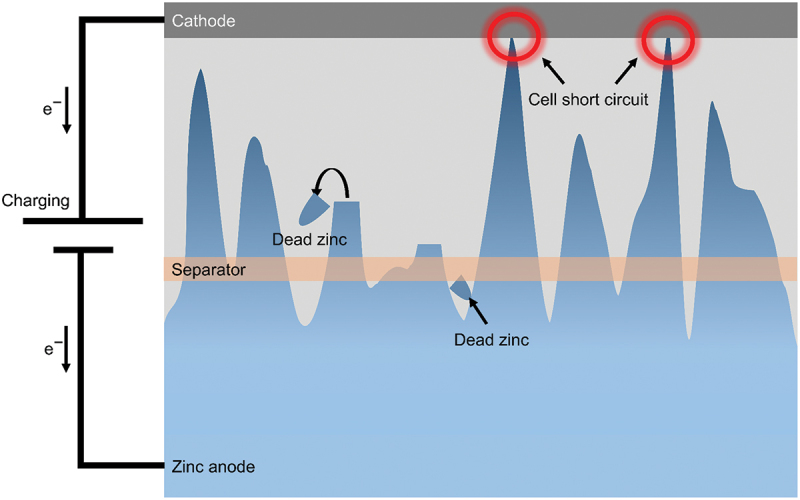
Schematic illustration of dead zinc formation and dendrite penetration through the separator that leads to internal short circuit due to excessive dendrite growth.

The term dead zinc refers to zinc dendrites which are generally considered inactive species that eventually detach from the anode surface. Dendrites are susceptible to detach from the zinc substrate due to poor adhesion and become inactive dead zinc [23]. The loose structure of dendrite lowers the contact area while also reducing electron transport at the interface. Zinc dendrites can grow up to 150 μm in length, considerably larger than the separator which is only 50 μm thick [10]. Through repeated cycle, these dendrites tend to penetrate the separator and make contact with the air cathode which poses a risk of short circuit as shown in Figure 5. Fortunately, there is no risk of explosion or combustion associated with zinc dendrite formation unlike LIBs [19]. Furthermore, in comparison to pure zinc metal, the higher surface energy of zinc dendrites may accelerate the production of unwanted hydrogen bubbles on the anode surface. Dendrite formation is mostly observed in alkaline electrolytes because of strong electrochemical activity by zinc metal [22].

Zinc dendrite is more serious under high current density because the growth rate is strongly associated with current density. Deposition rate of zinc is significantly influenced by the current density [37]. The quality of zinc deposition is degraded with higher current density due to increased mass transport requirement. To get this relationship, the Sand’s time, τ in the diffusion model is empirically related to the electron and zinc ion transport characteristics based on the following equation [38].

(2) $$\tau =\mathrm{\pi D}\frac{eC_{0}{\left(\mu_{a}+\mu_{Zn^{2+}}\right)}^{2}}{2J\mu_{a}}$$

where τ is the time when zinc dendrites start to grow, D is the diffusion coefficient, e is the electronic charge, C_0_ is the initial concentration in electrolyte, µ_a_ and µ_Zn2+_ are the anionic and Zn^2+^ mobility, respectively and J is the effective electrode current density. This equation is specifically applicable to neutral or mildly acidic electrolyte systems, where Zn^2+^ is the dominant species involved in diffusion and deposition. A higher τ is associated with smaller effective electrode current density (J) and increased Zn^2+^ ion mobility. This correlation suggests that the battery exhibits extended lifespan before the development of zinc dendrites [26]. The rationale behind this is that lower current density tends to result in more uniform distribution of local current densities, thereby reducing the distortion of surface electric fields. In order to minimize local current density, an increase in the specific surface area of the anode can be considered [39]. The behaviour of zincate ions in alkaline solutions has received significant attention for study because it can produce non-uniform dendrite growth which affects the performance of ZABs.

Dendrites accelerate the formation of passivation layer by promoting localized reactions. For this reason, ZABs experience high overpotentials. During operation, the formation of passivating films induces the transition from active dissolution to pseudo-passive state, restricting the efficient dissolution of zinc and elevating the overpotential [40]. This passivation effect results in substantial charge and discharge overpotentials, consequently reducing the energy efficiency. While theoretical equilibrium potential for zinc anodes is 1.65 V [41], achieving this potential in practical applications remains challenging. As the result, ZABs typically operate with discharge voltages below 1.2 V and charge voltages exceeding 2.0 V [42], leading to reduced practical capacity and diminished energy efficiency. In electrochemically rechargeable ZABs, achieving deep discharge of zinc anode is challenging because the zinc skeleton cannot be sufficiently maintained after reaching 100% depth of discharge (DOD). DOD refers to the proportion of total capacity that has been utilized during discharge cycle. For ZABs, DOD specifically indicates the extent to which the zinc anode has been oxidized during discharge process. 100% DOD implies complete utilization of available zinc, meaning all the active zinc material in the anode has been consumed in the electrochemical reaction. At DOD below 1%, the zinc anode oxidizes to generate a thin layer of ZnO which is below the critical passivation size of 2 µm [43]. The electrolyte effectively dissolves the small amount of zinc species formed during this discharge and allows the anode to refresh itself [44]. In the subsequent charge cycle, zinc deposition occurs smoothly on the anode surface, ensuring efficient charging and maintaining functionality. As DOD increases to 42%, a thick ZnO layer fully covers the zinc anode and form a substantial passivating shell. When the concentration of zincate ions exceeds saturation limit, ZnO would be precipitated on the surface of anode [10]. The passivation layer reaches an extensive thickness of up to 50 µm that exceed the critical size for passivation [44]. Therefore, not all the zinc oxide can be converted back into metallic zinc during the reduction process in charging. A significant portion of zinc remains electrically uncontacted due to the low electron conductivity of ZnO. The formation of thick passivation layer reduces the actual discharge capacity and lead to battery failure by preventing further discharge of internal fresh zinc metal. This passivation will block the transport of chemical species between electrodes and electrolytes. This problem generally worsens with the present of excess dendrite. As the passivation layer thickens on dendrite structure, the actual active area of zinc anode decreases and directly leads to an increase in anodic current density.

Dendritic growth on the anode side profoundly affects the performance of air cathode in ZAB systems. Dendrites protrude into the electrolyte and obstruct ion pathways which cause uneven current distribution. This disruption increases internal resistance of ZAB cell and elevates the overpotential required to sustain OER. Furthermore, inhomogeneity in ionic flux due to dendrites leads to non-uniform reaction rates that causes sluggish ORR and OER activities. Dendrite growth promotes parasitic reaction of zinc corrosion that competes with OER, thereby reducing overall energy efficiency of ZABs. The branching morphologies of dendrite changes the electrolyte composition near the electrode interfaces [45]. Localized depletion of OH^−^ significantly hinder the kinetics of ORR and OER. In alkaline systems, these reactions are highly sensitive to alkalinity [46]. Lower OH^−^ concentrations result in diminished catalytic activity and require higher overpotentials to sustain the reactions [47]. Li et al. [48] justified that depletion of OH^−^ creates strong spatial electric field that influences the deposition and further reduces the local concentration of OH^−^ ions.

### Hydrogen evolution reaction

**Figure 6.**
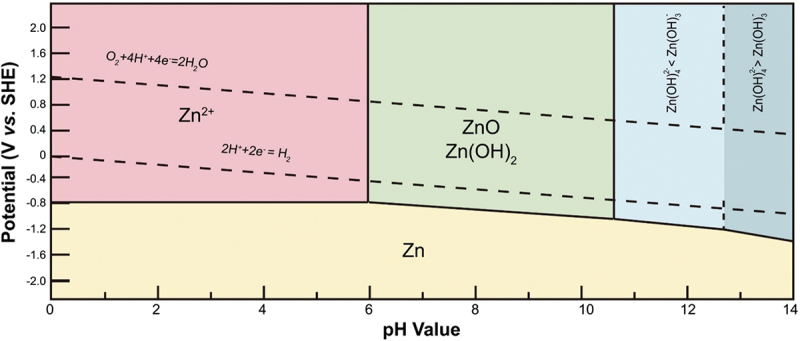
The Pourbaix diagram of Zn in aqueous solution. Reproduced with permission from ref [19]. copyright © 2021 Elsevier.

Ion migration occurs across the medium of electrolyte. It has significant effect on the discharge potential, rechargeability and overall performance of the cell. KOH and NaOH are frequently used as aqueous electrolyte in ZABs due to the desirable ionic conductivity. High concentration electrolytes have been developed to enhance the performance of ZABs by regulating the interaction between Zn^2+^ ions and water. As the electrolyte concentration increases, the availability of free water molecules decreases, thereby suppressing hydrogen evolution reaction (HER) and corrosion phenomena associated with water [49]. Throughout zinc dissolution and deposition process, the pH of electrolyte has a significant effect on the intermediate Zn species produced [50]. Figure 6 shows the Pourbaix diagram that is used to predict the stability of different species under varying conditions of pH. Depending on the pH of electrolyte, different species of zinc are formed [19]. In strong alkaline condition (pH > 12): Zn + _4_OH^−^ ⇌ Zn(OH)_4_^2¯^ + 2e¯, in mildly alkaline condition (pH = 8 to 12): Zn + 3OH^−^ ⇌ Zn(OH)^3¯^ + 2e¯ and in near-neutral condition (pH < 8): Zn + 2OH^−^ ⇌ Zn(OH)_2_ + 2e¯. Therefore, electrolyte environment influences the mechanism and equilibrium potential of zinc anode reactions.

**Figure 7.**
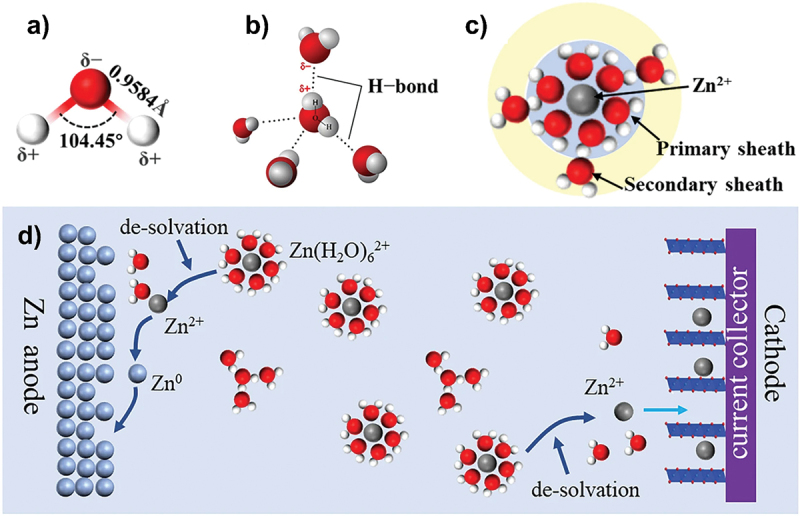
Schematic representation of (a) chemical structure of water, (b) hydrogen bond formed water clusters, (c) hydrated structure of zinc ions in diluted aqueous electrolytes and (d) arrangement of water molecules and zinc ions in the electrolyte. Reproduced with permission from ref [51]. copyright © 2023 Springer nature.

Aqueous electrolyte contains water with different configurations such as water clusters and single water molecule: (H_2_O)_n_ → (H_2_O)_n-1_ + H_2_O [51]. Multiple water molecules can indeed form water clusters ((H_2_O)_n_) through hydrogen bonding. When soluble salts are added to the solution, the original hydrogen bond network in water may be disrupted as ions from the salt interact with water molecules. Specifically, when zinc ions are added, they do not exist as free ions but instead coordinate with water molecules to form hydrated zinc ions (Zn(H_2_O)_6_^2+^). The coordination involves electrostatic attraction between positively charged zinc ions and negatively charged oxygen atoms of water molecules due to the polar nature of water as shown in Figure 7a–c. Sheath structure of Zn(H_2_O)_6_^2+^ is not conducive to the migration and deposition of zinc ions which lowers battery performance [52]. Based on Figure 7d, during charging process, hydrated zinc ions undergo desolvation into zinc ions and water molecules at the anode interface: Zn(H_2_O)_6_^2+^ → Zn^2+^ +6 H_2_O. However, the presence of independent free water molecules can lead to poor thermodynamic stability and this can contribute to parasitic reactions on the anode interfaces [51]. These water molecules may hydrolyse and produce H^+^ ions which can be further reduced to form hydrogen gas. Moreover, ionization equilibrium of free water also generates H^+^ and OH^−^ (H_2_O → H^+^ + OH^−^). As zinc electrode has more negative potential, water molecules are more likely to obtain electrons on the zinc metal surface, leading to the generation of H₂ gas (2H^+^ + 2e^−^ → H_2_) and OH^−^ (2H_2_O + 2e^−^ → H_2_ + 2OH^−^) [53]. This set of reactions can occur not only during charging process but also during idling processes of ZABs. Faraday efficiency of zinc dissolution would be significantly reduced due to the presence of HER. The consumption of zinc and electrolyte by the side effect of HER severely reduces discharge capacity because zinc is the only active species that produce electric energy [54]. This reaction is also irreversible. Furthermore, the strong adsorption of hydrogen bubbles on the zinc surface has prevents ions exchange between the zinc anode and electrolyte [10]. Apart from that, safety issues such as battery pack swelling, potential leakage and explosion may occur due to accumulation of large amount of hydrogen gas in close system. HER increase internal pressure and causes the sealing to fail. However, there is no such concern in the case of ZABs because they are open systems.

During discharge process, spontaneous chemical reaction occurs between zinc and OH- to produce ZnO. The problem of zinc anode is closely related to electrolyte because zinc metal is highly reactive towards OH-. When two electrons are released, the electrical stripping of zinc metal into electrolyte during discharge process is referred to as the zinc corrosion or parasitic reaction [10]. This corrosion reaction on the anode side occurs spontaneously in the absence of external loads. This process is known as self-discharge and it would significantly shorten the operational life when the air electrode had access to oxygen. Parasitic self-discharge reaction occurs due to low thermodynamic stability in the aqueous solution and produce hydrogen gas [55]. Importantly, both zinc dissolution and zinc corrosion have identical potentials. Therefore, the electrons lost by zinc metal may selectively interact with water and result in the creation of hydrogen (Zn + 2 H_2_O + 2OH^−^ → Zn(OH)_4_^2−^ + H_2_).

HER at the anode side produces very fine hydrogen bubbles that migrate and diffuse toward the air cathode, where they interfere with critical processes of oxygen reduction [56]. The presence of hydrogen bubbles in gas diffusion layer (GDL) reduces the availability of reactants necessary for ORR and subsequently lowers the efficiency of ZABs [57]. This not only diminishes the rate of ORR but also reduces the effective surface area available for reaction, limiting the number of active sites and further impairing ZABs performance [58]. The intrusion of hydrogen bubbles into GDL can form gas chambers, disrupting the designed gas transport structure [59,60]. This interference affects electrolyte distribution, leading to inadequate contact between the electrolyte and catalyst in the catalytic active layer of ZABs. As the result, the kinetics of both ORR and OER are compromised due to insufficient electrolyte–catalyst interaction. Additionally, the movement of hydrogen bubbles induces local convection within the diffusion layer, enhancing mass transport of zincate ions [61]. This electrolyte displacement in ZAB system increases the risk of saturation within GDL, overwhelming the hydrophobicity [62]. The hydrophobicity of GDL is engineered to repel water and maintain efficient gas pathways. However, continuous exposure to hydrogen bubbles can disrupt this hydrophobic balance. Over time, this leads to increased water accumulation and localized flooding within the GDL. Such flooding destroys the structural integrity of GDL, severely impacting overall ZABs performance.

## Zinc anode modifications

**Figure 8.**
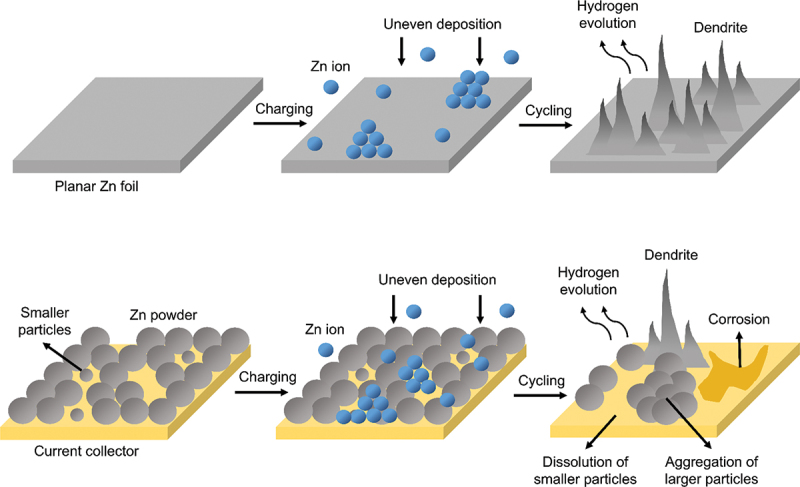
Illustration of planar and powder zinc anode with dendritic morphologies during cycling due to uneven zinc deposition.

The performance of ZABs is hindered by limitation in the surface structure and low utilization efficiency of zinc anode [4]. Based on Figure 8, two main types of anodes which are powder and planar zinc anode have been previously used [63]. Powder-bed anode involves compacting zinc powder into a bed-like structure. This design incorporates finely divided zinc particles, usually mixed with conductive additives and binders to enhance electrochemical performance and structural integrity. However, the electrochemical performance of conventional polymer-binder-composite zinc powder anode has been reduced due to limited ion and electron transfer, along with electrical contact failure caused by volume effects [64]. Zinc powder anode tends to develop dendritic morphologies during cycling which increase the susceptibility to corrosion due to the presence of rough spherical surfaces [65]. In addition, uneven particle size distribution promotes dendrite growth due to the aggregation of larger particles [66]. On the other hand, planar zinc foil offers simpler design for the anode which act as both current collector and active material [67]. This foil has good electrical conductivity and provide stable platform for the deposition and stripping of zinc ions during charge and discharge cycles. However, small reactive surface area and limited nucleation sites of bare planar zinc anode cause severe dendritic growth [68]. Planar zinc foil inherently demonstrates low wettability, surface passivation and restricted ion and electron transport routes which impede the uniform deposition of metallic zinc [69].

To address these challenges, this section provides comprehensive discussion of anode modifications, specifically focus on alloying, nanoporous structures and zincophilic surface treatments as potential solutions, which are particularly relevant to ZABs. Given that all zinc-based energy storage systems share similar anode-related challenges due to similar electrochemical processes and conditions within aqueous electrolytes, the proposed anode modifications are broadly applicable across these systems. This approach not only informs the development of ZABs but also provides valuable insights and directions drawn from the collective body of work in the broader field of zinc-based batteries.

### Alloying zinc anode

**Figure 9.**
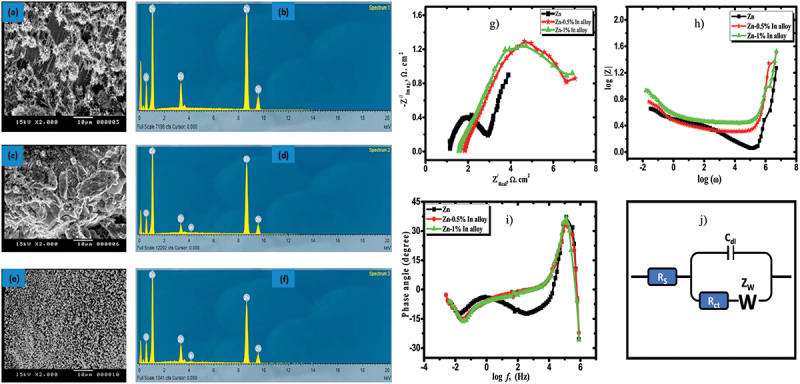
SEM images and EDAX analysis of the oxide layer formed on a-b) Zn, (c−d) Zn-0.5% in alloy and (e−f) Zn–1%In surfaces at the active region. (g) Nyquist plot, (h) bode plot, (i) bode phase and (j) equivalent circuit of bare-zn and Zn–in alloys measured at AC amplitude of 10 mV with frequencies ranging from 10 kHz to 10 mHz in 6 M KOH solution at 25°C. Reproduced with permission from ref [75]. copyright © 2021 Elsevier.

Zinc metal anode can be alloyed to form binary, ternary and multi-element alloys. When alloy components interact, it can form two primary types of phases, which are solid solutions and intermetallic compounds [52]. Each phase has different structures and properties which depends on the specific alloying elements and composition. It can be optimized by adding the right proportion of alloying element by referring the phase diagram. The presence of alloying elements influences the interaction between anode and electrolyte, which in turn supports high discharge stability and specific capacity of ZABs [70]. A key study by Li et al. revealed that developing multiphase Cu-Zn alloy interlayer to the surface of anode effectively prevents zinc atoms from clumping together and reduces unwanted side reactions such as corrosion [71]. Admittedly, Meng et al. demonstrated that the formation of Zn_x_Cu_y_ alloy shell facilitates even zinc deposition with low nucleation overpotential as low as zero millivolt [72]. Apart from that, during charge-discharge process, zinc undergoes surface alloying and strong metallic bond is formed within the alloy. The high binding energy of this bond improves chemical stability of the anode surface. Tian et al. demonstrated that Sb exhibits excellent alloying reaction by forming ZnSb intermetallic compound during discharge [73]. The reaction Sb + xZn ↔ Zn_x_Sb confirms the feasibility of using Sb as a reversible alloying anode for zinc-ion batteries. This approach not only strengthens the anode but also helps prevent dendrite formation. Elrouby et al. [74] stressed the time required for passivation increases with the addition of Sb and the energy efficiency is enhanced with Zn-0.5%Sb alloy. Electrochemical impedance spectroscopy results show the resistivity of charge transfer and Warburg impedance decrease, while the double-layer capacitance increases with Sb addition, indicating improved electrochemical performance in alkaline media. Similarly, Zn – In alloys show the double-layer capacitance increase with higher content of indium up to 1% wt (Figure 9) [75].

**Figure 10.**
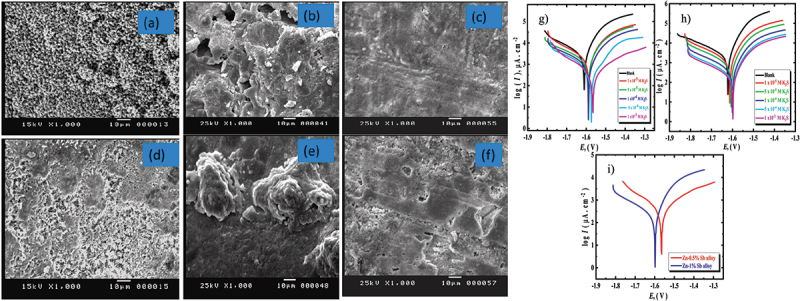
SEM images for alloyed zinc with (a-c) 0.5% Sb and (d-f) 1% Sb in the KOH electrolyte. Tafel curves of alloyed zinc with g) 0.5%sb and h) 1%sb in the KOH solution containing different concentrations of potassium sulphide at temperature 25°C and i) comparison between the two studied alloys. Reproduced with permission from ref [76]. copyright © 2024 Elsevier.

**Figure 11.**
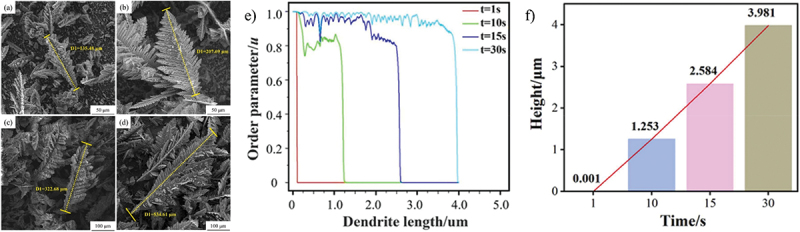
Evolution of zinc dendrite formation with time. Constant-current charge for (a) 5 min, (b) 8 min, (c) 15 min and (d) 20 min. (e-f) dendrite growth height at different charging times. Reproduced with permission from ref [79]. copyright © 2024 Elsevier.

The structure of compound in alloys differs from that of pure zinc anode [4]. Therefore, some properties are changed such as corrosion resistance, chemical state and electrical conductivity. For example, the corrosion current decreases as Sb alloying content increase from 0.5%Sb to 1%Sb in the examined KOH solution, resulting in thick porous layer of the product of corrosion [76] (Figure 10). Indeed, alloying modification can change the surface properties and electronic states [77]. It lowers the nucleation barrier for zinc deposition during charging process. Furthermore, the presence of alloying element such as Ga, Sn, Cu, P, and Al can act as the 3D current collector that provide high stability and prevent dendrite formation [78]. These alloys have high electronic conductivity and enlarged electroactive surfaces which help suppress dendritic growth and hydrogen evolution reactions. Additionally, a study by Yuan et al. found the addition of Ce, Yb and Mg resulted in 35% reduction in dendrite growth [79] (Figure 11). Admittedly, He et al. mentioned the eutectic Zn-Yb alloy refines the grains of Zn anode, smoothing the surface and reducing ion diffusion resistance [80]. The addition of these rare earth elements lead to the formation of dense and compact corrosion product layer, which impact the overall electrochemical environment of ZABs [79]. However, the practical application is limited by the high cost and limited availability of rare earth elements like Ce and Yb.

Furthermore, the addition of Al and In constituent has been shown to effectively control HER and reduce corrosion in ZABs. Specifically, zinc alloy fabricated with 3 wt% In (ZI3) demonstrated significant improvements with high specific discharge capacity and large capacity retention [81]. The combination of In, Bi and Ca in zinc alloy anode increases corrosion resistance and improves discharge performance by inhibiting the formation of ZnO passivation film [82]. Moreover, incorporating Bi into zinc anode improves the morphology of oxide film and enhances mass transfer between the solid–liquid interfaces [83]. Likewise, Zn-Bi alloy with 2 wt% bismuth achieved discharge capacity retention of 99.50% in ZABs. The addition of indium has been shown to retard the dissolution of zinc in active region and reduce passivation time. Indium alloying also eliminates high oscillations in potential observed with pure zinc, leading to more stable electrochemical behaviour in alkaline environment [84]. The polarization resistance and Warburg impedance increase, while the double-layer capacitance decreases with indium addition, indicating improved passivation resistance and energy efficiency [85].

**Figure 12.**
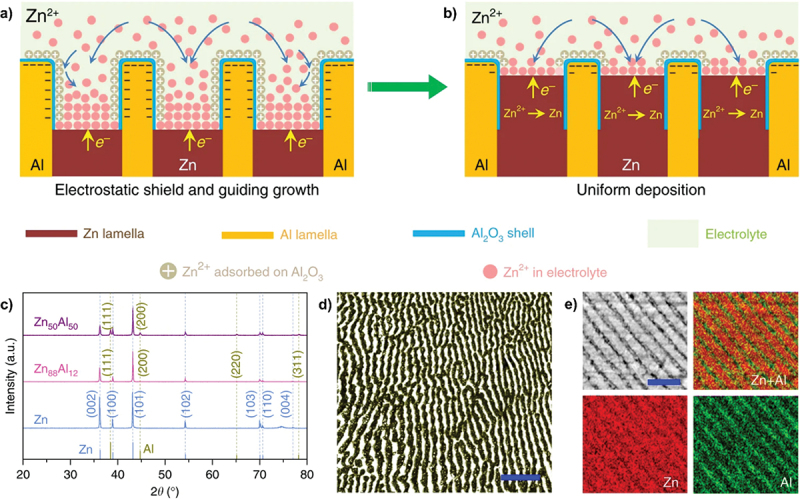
Schematic illustration and characterization of lamellar structure. (a) eutectic lamellar structure of Zn-al alloys that consists of alternating Zn and Al nanolamellas, generate core/shell interlayer patterns in-situ during Zn stripping to help direct subsequent Zn plating. (b) uniform Zn deposition by the Al/Al_2_O_3_ interlayer patterns. (c) XRD patterns, (d) optical micrographs with 10 μm scale bar and (e) SEM image with 2 μm scale bar and corresponding EDS element mapping of eutectic Zn_88_Al_12_ alloys. Reproduced with permission from ref [86]. copyright © 2020 Springer nature.

**Figure 13.**
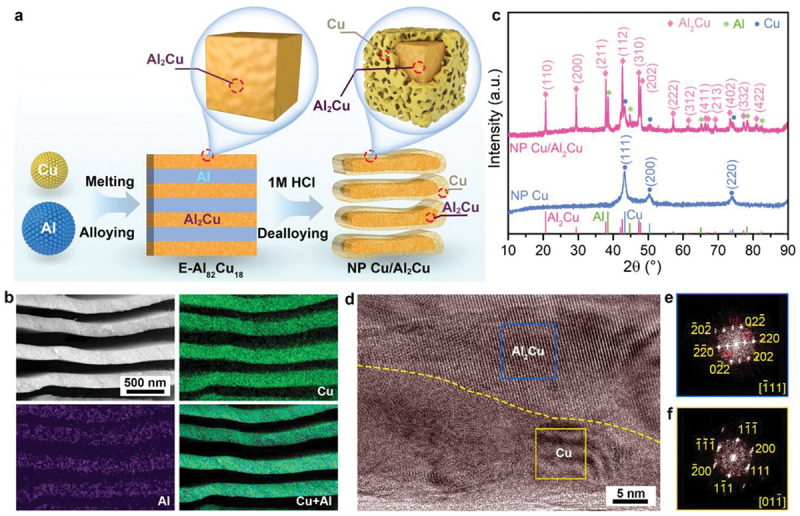
Fabrication process and characterization of the nanoporous lamellar anode. (a) schematic illustration of the fabrication process for the lamellar NP Cu/Al₂Cu metal/intermetallic compound heterostructure anode via alloying and chemical dealloying. (b) SEM image along with EDS elemental mapping, (c) XRD patterns, (d) HRTEM image of the NP Cu/Al₂Cu heterostructure and (e,f) FFT patterns of selected regions at the Cu/Al₂Cu interface in (d). Reproduced with permission from ref [89]. copyright © 2024 John Wiley and sons.

Interestingly, a significant study by Wang et al. introduced alternating lamellar-nanostructured eutectic Zn_88_Al_12_ alloy as a dendrite-free anode as shown in Figure 12 [86]. The uniformly ordered lamellar pattern of Zn and Al phase is further confirmed by XRD and SEM with the corresponding EDS element mapping. The formation of Al_2_O_3_ shell on the Al lamellar protects against the dissolution of Al. This Al_2_O_3_ shell prevents Zn dendrite formation by guiding electrodeposition onto precursor Zn site and avoids zinc ions accumulation through electrostatic shielding mechanism [87]. Correspondingly, the presence of Al also protects against the formation of irreversible byproducts such as ZnO and Zn(OH)_2_ within the anode surface because Al is preferentially oxidized to form Al_2_O_3_ [88]. Consequently, this reduces passivation of zinc anode in alkaline system. Similarly, Meng et al. proposed the layered nanoporous lamellar structure of Cu/Al_2_Cu as shown in Figure 13 [89]. Due to local galvanic couples, the interconnected ligaments enable fast electron transfer and provide highly zincophilic sites for zinc nucleation, while the interpenetrating lamellar channels act as mass transport pathways. As a result, it shows remarkable performance with extended 4000 h lifespan at 0.5 mAh/cm^2^. This modification is set to become a new type of Zn-free anode, providing innovative approach for high performance ZABs. However, creating nanostructured alloy involves complex requirements [90]. Achieving high specific capacity requires precise adjustment of the active material in the alloy [91].

Although alloying anode can prevent dendritic growth and minimize side reaction, but Zn alloys with more active metals than Zn itself face issues such as corrosion and competitive reaction among metal species [52]. For Zn-Ni alloy, Ni has strong hydrogen evolution activity, causing poor Zn deposition process in alkaline ZABs [77]. The factors like element ratio, microstructure and alloy phase affect the corrosion resistance of alloys that make it challenging to fully optimize this property [52]. Due to limited research and lack of comprehensive database, the effect and cost implication of using different metal in Zn alloy electrode remain poorly understood [23]. Additionally, alloy preparation methods such as electrodeposition and chemical replacement often result in limited and irregular structures. The addition of alloying element can further increase manufacturing costs and complexity, potentially hindering large-scale commercialization.

### Nanoporous design

ZABs are highly versatile energy storage systems with applications ranging from compact button cells to large-scale grid storage. The adaptability of porous materials which can be engineered in diverse forms such as bulk monoliths [92], melt-spun ribbons [93], compressed metallic powders [94], foams [95] and sponges [96] offers significant advantages in tailoring electrode structures to meet specific functional and structural requirements. The inherent tunability of porous materials enables the optimization of key parameters such as surface area, porosity and mechanical robustness, providing unparalleled flexibility for ZAB design. Indeed, nanoporous electrode has shown great potential for energy storage applications due to the special characteristics including low relative density, high surface area and excellent permeability [97]. It is valuable not only for ZABs but also for supercapacitor, fuel cell, catalysis, actuator, sensor and bio-device [98]. In the context of ZABs, nanoporous electrode offers many advantages over standard powder-bed and planar zinc electrodes, especially in minimizing the resistances encountered during charge and discharge process such as ion transport, electrochemical reaction and electron conduction in the electrode. Nanoporous structure also balance the distribution of electric field, suppress dendrite formation and lower the local current density due to larger reactive surface area [99]. It contributes to maintain optimal ionic conditions, indirectly benefiting the air cathode by preserving consistent reactant availability [100].

Nanoporous surface provide ligament frameworks for fast electron conduction and open-pore channels for rapid mass transport. Bicontinuous network is formed when pore channels and solid ligaments are interconnected with each other [101]. It has several advantages including large surface-to-volume ratio, good electrical conductivity, abundant surface defect and high mechanical strength [102]. Anode with nanoporous design can reduce the four principal resistances which are [1] ion transport in the electrolyte [2], ion transport in the anode [3], electrochemical reactions in the anode and [4] electron conduction in the anode and current collector [63]. This contributes to minimize large overpotential of ZABs due to sluggish OER and ORR at the air cathode side. When measuring the length and diameter distributions of pore and ligament, they are closely related which is morphologically equivalent while having opposite characteristics of one another in three dimensions [98]. For example, if the ligaments are thin, the pores would be wider and vice versa. Along with porosity, the concept of tortuosity is utilized to evaluate the effective ion diffusion coefficient. Tortuosity refers to the irregular and convoluted paths that ions must navigate within the pores, which can significantly impact the rate of diffusion [103]. Although porous design offers good functional properties on the surface, it has limitation in the electrolytic ion transport and storage due to tortuous diffusion paths [104]. However, hierarchical architecture of nanoporous anode can further increase the mass transfer efficiency by using large pores as a shortcut to transport [97]. Integrating multiscale pores with combination of nano-sized and micron-sized pores can improve the transport properties. Porous materials are classified into three categories based on pore size which are microporous (<2 nm), mesoporous (2–50 nm) and macroporous (>50 nm) as defined by the International Union of Pure and Applied Chemistry (IUPAC). Hierarchically constructed porous materials consists multiple length scales from micropores to mesopores and macropores. Micropores play important role in reducing material tortuosity and increase effective diffusivity while nanopores increase the specific surface area [105].

Several methods such as dealloying, electrochemical deposition and templating was used to synthesis porous surface. Electrode with high porosity offer many benefits, even though the porous electrode itself may not directly participate in redox reactions. When active materials are deposited onto inert nanoporous electrode, it enhances the performance of ZABs by strengthening electric conductivity, improving ion diffusion and regulating volume fluctuations [98]. For example, carbon-based porous electrode like graphite felt and carbon cloth offer high electrical conductivity and stability. 3D porous graphene has better mesoporosity with good electron and ion transport network compared to conventional 3D reduced graphene oxide (3D-rGO) [106].

**Figure 14.**
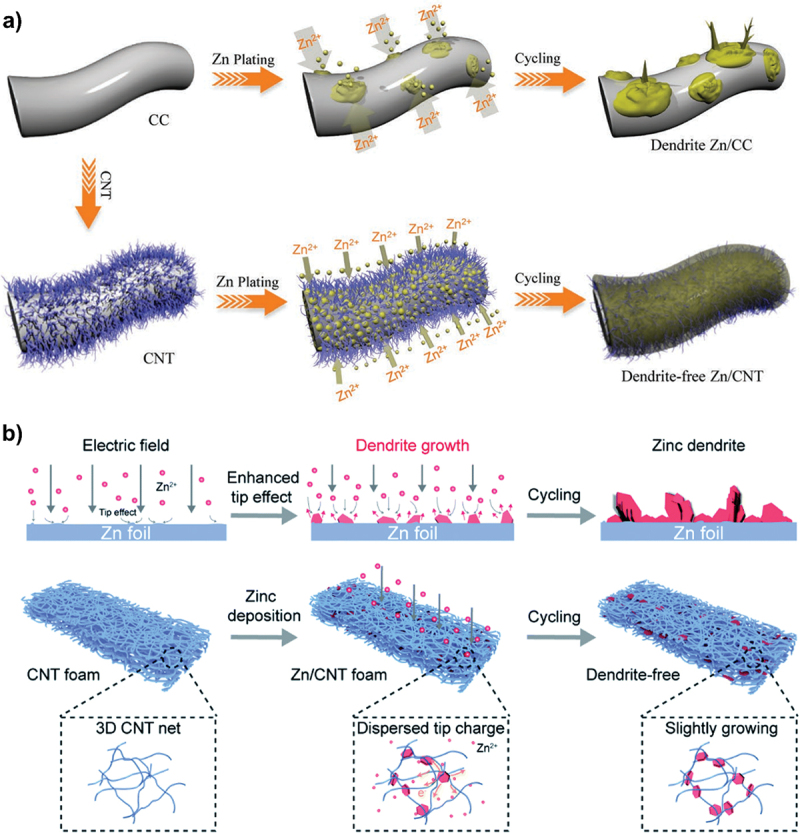
Schematic illustration of zinc plating and cycling on (a) dendrite-free Zn/CNT anode and b) Zn/CNT foam anode. a) reproduced with permission from ref [107]. copyright © 2019 John Wiley and sons. (b) reproduced with permission from ref [108]. copyright © 2020 the royal society of chemistry.

**Figure 15.**
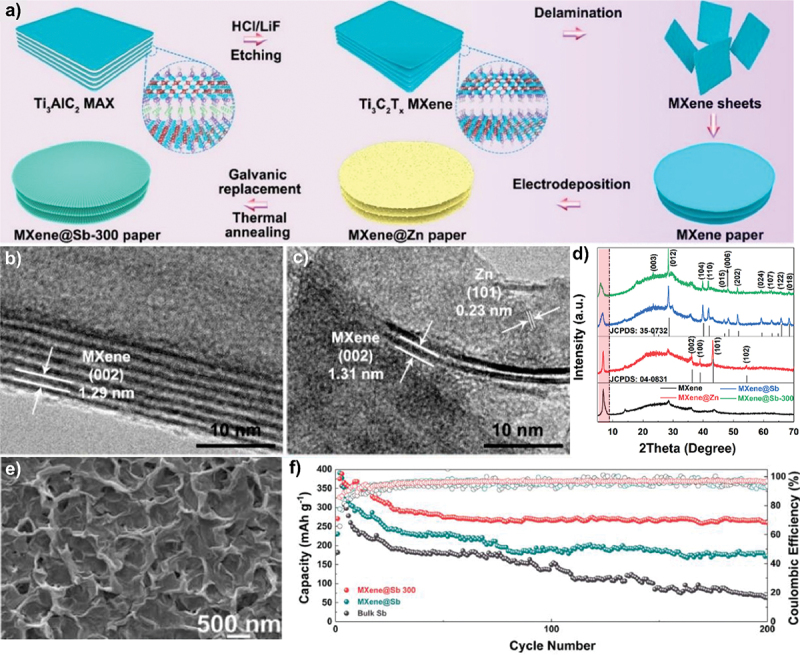
Fabrication process and characterisation of zinc-based anode supported on MXene paper. (a) schematic illustration of fabrication process of MXene@Zn paper, high resolution TEM of (b) Ti_3_C_2_T_x_ MXene and (c) MXene@Zn, d) XRD patterns, (e) SEM image of MXene@Sb-300 anode after 200 cycles at 0.1 mAh/cm^2^. Reproduced with permission from ref [73]. copyright © 2021 Elsevier.

**Figure 16.**
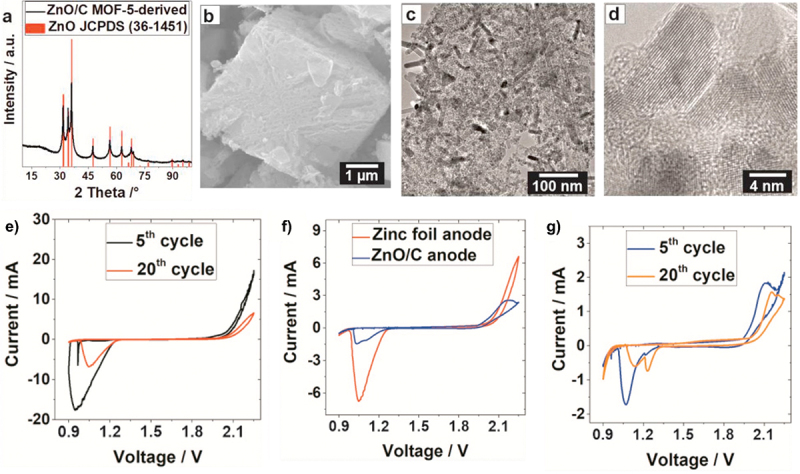
Characterization of MOF-5 derived ZnO/C composite. (a) XRD pattern, (b) SEM image, (c) morphology with nanorod and spherical ZnO nanoparticle composition, (d) HRTEM image of crystalline ZnO particles embedded in amorphous carbon matrix. (e) CV curve of zinc foil anode in alkaline ZAB full cell. (f) comparison of CV curve for zinc foil anode and ZnO/C anode. (g) two different zincate species are identified in the CV diagram of ZnO/C composite anode. Reproduced with permission from ref [109]. copyright © 2021 Elsevier.

**Figure 17.**
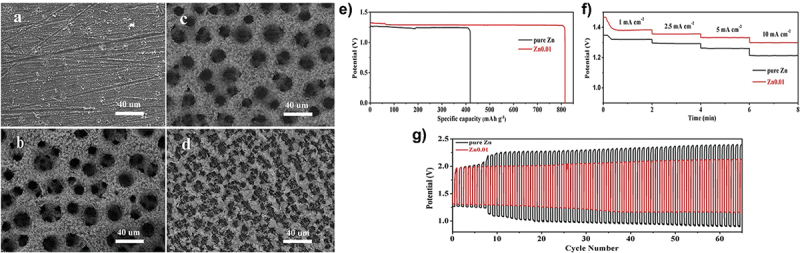
(a-d) multi-scale porous structures with smaller pores formed on the walls of larger pores. (e) ZAB discharge tests at 5 mA/cm^2^, (f) discharge tests at different current densities, (c) discharge-charge tests at 5 mA/cm^2^. Reproduced with permission from ref [110]. copyright © 2019 frontiers.

In addition, carbon nanotubes (CNTs) have been employed as electron and ion redistributors to provide large active surface area [67]. As shown in Figure 14a, Zn^2 +^ ions tend to deposit on protruding sites during cycling which leads to dendrite formation. The incorporation of 3D CNT scaffold significantly improves the uniformity of electric field distribution due to its increased surface area from the 3D structure. This CNT structure promotes more uniform ion deposition. Apart from that, the CNTs design allows optimal use of both surface and interior regions which increases the overall active surface area (Figure 14b). Therefore, the use of CNTs holds great potential as a host material for dendrite-free zinc deposition. Furthermore, Tian et al. designed zinc-based anode supported on MXene paper using controlled electrodeposition method as shown in Figure 15 [73]. The expanded interlayer spacing within MXene layers increases ion accessibility and promotes the formation of more electroactive sites. The MXene@Sb-300 anode demonstrates excellent cycling performance and retains capacity of 299.6 mAh g^− 1^ at 50 mA g^− 1^ after 200 cycles. This performance is attributed to the architecture of MXene which provides additional active sites for electrochemical reactions. Additionally, Figure 15e shows smooth surface on MXene@Sb-300 anode even after 200 cycles and confirms the absence of dendrite formation. Apart from that, porous framework of MOF-5 structure enhances zinc accessibility through its unique 3D architecture, promoting better zinc utilization. MOF-5 shows improved electrochemical performance in ZABs compared to the planar zinc anode, with efficient ZnO reduction and minimal passivation [109]. The stable zincate formation peaks further indicate improved reaction kinetics, making the composite anode more reliable choice for ZAB applications (Figure 16). Besides that, multi-scale porous structures outperform pure zinc in alkaline ZABs, exhibiting higher discharge capacity and enhanced cycling stability. It maintains smaller charge-discharge voltage gap (0.63 V vs. 1.02 V) and demonstrates longer cycle life with stable discharge voltage up to 33 cycle, compared to the decline observed in pure Zn after 8 cycles (Figure 17) [110].

While these advancements can improve the performance of anode, it come with disadvantages such as complicated fabrication process and high cost. Admittedly, surface modifications such as thermal treatment and the growth of CNTs involved complex synthesis processes that are impractical for large-scale manufacturing [68]. Apart from that, the primary challenge remains such as nonuniform zinc nucleation and dendritic deposition resulting from the low binding energy of carbon materials with zinc ions. For porous graphene nanomaterials, achieving uniform pore size, structure and morphology requires precise control of synthesis conditions. This includes the use of specific preparation techniques such as hydrothermal, chemical activation, thermal exfoliations, chemical vapour deposition and template approach [111]. Furthermore, the production process involves multiple stages and various raw materials, often leading to low carbon yield. Alternatively, porous metal frameworks such as Cu foam have emerged as another promising host for zinc anodes due to their enhanced zincophilicity, high porosity and excellent electrical conductivity [112]. However, the host material does not directly participate in redox reactions during charge and discharge processes. Therefore, it results in limited energy density. Incorporating foreign materials reduce the proportion of high-capacity active material in the anode, thereby reducing the overall specific capacity.

Although nanoporous design shows potential, there is still room for improvement. Further optimization is needed to ensure rapid ion and electron transmission, as well as homogeneous interface and chemical distributions at the nanoscale surface porosity [113]. It is important to note that increasing the surface area can lead to elevated side reactions including the hydrogen evolution reaction and zinc self-corrosion which can result in low coulombic efficiency and rapid capacity degradation. As a result, the optimal nanoporous surface for zinc anode must be carefully designed for ZAB system.

### Zincophilic design

In ZAB system, zincophilic design plays critical role in optimizing the utilization of available zinc ions. In open systems where high atmospheric humidity can lead to electrolyte absorption, excess water may promote undesired HER which compromises battery efficiency [114]. Zincophilic properties mitigate these effects by promoting selective zinc deposition, thereby suppressing competing HER reactions that could otherwise degrade performance. Furthermore, zincophilic surfaces enhance the stability of anode-electrolyte interface, reducing the sensitivity of open system to environmental fluctuations. Although anode-free strategies for ZABs have been discovered, the challenge still remains regarding the nucleation sites distribution and energy barrier on the surface of substrate [115]. As discussed earlier, zinc deposition involves four critical steps which are mass transport, desolvation, nucleation and crystal growth. During nucleation process, poorly distributed and limited nucleation sites promote the formation of zinc dendrite. Based on the principles of heterogeneous nucleation theory, more compact and uniform zinc deposition can be achieved by controlling the adsorption and bonding ability of the interface to zinc ions [116,117]. For this reason, there is interest in developing advanced strategy through the modification of anode by introducing zincophilic environment. Zincophilicity refers to the affinity or attraction of molecule towards zinc ions which plays important role in electrochemical activity. Zincophilic surface lowers the energy barrier for zinc ion deposition and encourages more controlled zinc growth [99]. Moreover, rich zincophilic environment reduces the nucleation overpotential by providing more abundant electroactive sites to induce homogenous zinc deposition [118]. This is because the wide distribution of zincophilic sites promotes the formation of spacious Zn nuclei [119]. In addition, zincophilic surface also improves the wettability of electrode with the aqueous electrolyte and further prevents crystalline defects [120]. The integration of zincophilic species into the anode structure offers a wide range of benefits in term of interfacial properties [121,122]. Nevertheless, there is a trade-off between dendrite formation and unwanted side reaction when considering between zincophilicity and zincophobicity [123]. Zincophilic anode has the capability to homogenize zinc deposition and alleviate dendrite growth, meanwhile, zincophobic anode can prevent parasitic side reaction by regulating zinc ion solvation structure and suppressing water decomposition. Xie et al. designed the barium titanate protective layer as a zincophobic coating for zinc metal anodes [124]. They discovered that the weak interaction between Ba^2+^ ions and zinc atoms effectively guided zinc deposition beneath the protective layer. This weak bonding allowed zinc to preferably nucleate and grow across the anode surface. However, it presents major drawback of limited depth of discharge due to the restricted guiding effect on zinc ions [123]. Furthermore, the sluggish deposition kinetics of zinc ions caused by the insulating nature of zincophobic materials may result in higher voltage hysteresis and inadequate durability. Therefore, zincophilicity is considered as the most suitable characteristic for zinc anode.

**Figure 18.**
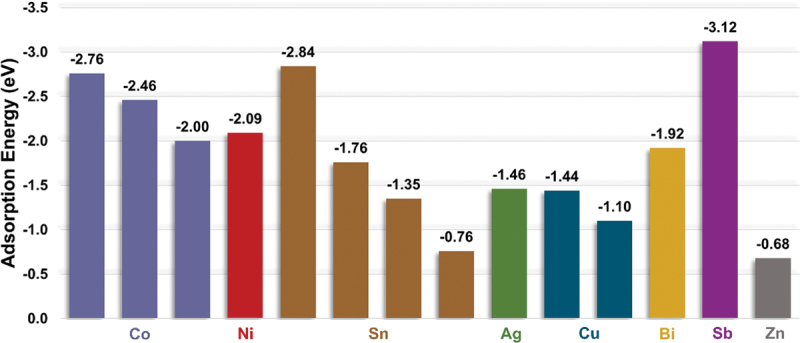
Adsorption energy between zinc atoms and various materials with zincophilic behaviour.

Various materials including cobalt (Co), nickel (Ni), copper (Cu), silver (Ag), tin (Sn), gold (Au), bismuth (Bi), indium (In) and antimony (Sb) have been investigated for their potential to create a zincophilic environment based on their high affinity for zinc ions. These materials offer zincophilic behaviours that readily capture zinc ions from the electrolyte. Once captured, zinc ions undergo reactions with electrons and lead to the formation of zinc atoms or clusters. This process effectively reduces the accumulation of zinc ions at the interfaces between anode and electrolyte. Figure 18 summarizes the adsorption energy between several materials and zinc atoms. Based on the following equation, density functional theory (DFT) can be applied to systematically calculate the adsorption energy (E_ads_) of zinc atoms on a substrate in order to quantify the affinity.

(3) $${\text{E}}_{\text{ads}}{\text{=E}}_{{\text{sub-}}_{\text{Zn}}}{\text{-E}}_{\text{sub}}{\text{-E}}_{\text{Zn}}$$

where E_sub-Zn_ is the energy of zinc adsorbed onto the substrate, E_sub_ is the energy of clean metal surface and E_Zn_ is the energy of isolated zinc atom [125]. A substrate is considered as zincophilic if the calculated E_ads_ is more negative than E_ads_ of a zinc atom on zinc metal which is −0.68 eV [126]. Based on Figure 18, antimony has the highest adsorption energy which is −3.12 eV. This indicates a strong attraction between zinc and antimony substrate which facilitates uniform zinc deposition. Besides atomic-level prediction using DFT computation, X-ray photoelectron spectroscopy (XPS) can be utilized to evaluate the energy level of electron. XPS test provide experimental result by observing shift in the spectra towards higher binding energy that indicate a strong interaction of zinc ions [127].

**Figure 19.**
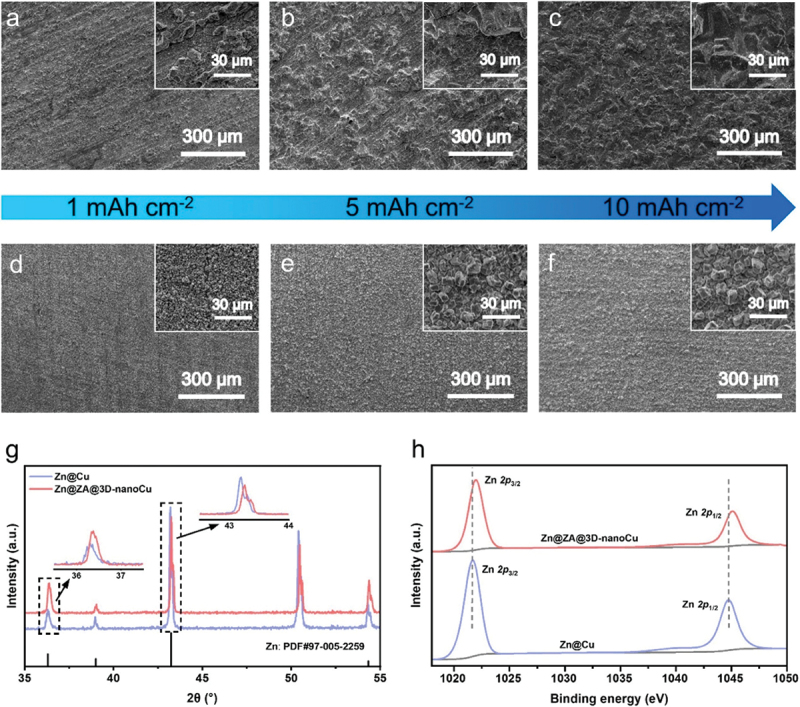
Zn deposition characterisation on Cu and ZA@3D-nanoCu substrate. SEM images of (a-c) Cu foil and (d-f) ZA@3D-nanoCu substrate plated with Zn with areal capacities from 1 to 10 mAh cm^−2^, (g) XRD patterns and h) Zn 2p XPS spectra. Reproduced with permission from ref [115]. copyright © 2023 Elsevier.

**Figure 20.**
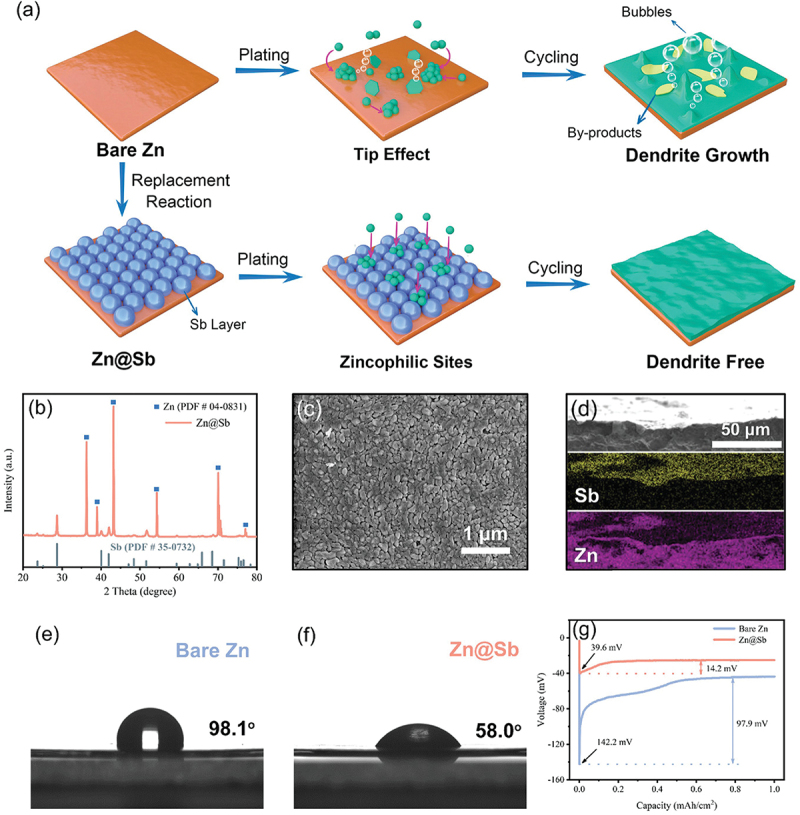
(a) schematic illustration of zinc plating and cycling on bare Zn and Zn@Sb, (b) XRD patterns, (c) SEM image, (d) cross-sectional SEM image and the corresponding elemental mapping of Zn@Sb. Contact angle measurement of ZnSO_4_ electrolyte on (e) bare zinc and f) Zn@Sb. (g) nucleation overpotential at 1.0 mA/cm^2^. Reproduced with permission from ref [129]. copyright © 2022 John Wiley and sons.

Continued research efforts are currently focused on optimizing the zincophilic design. In a recent study by Xu et al. [115], zincophilic Sb nanoparticles uniformly dispersed on 3D nano-copper substrate (ZA@3D-nanoCu) was investigated. As shown in Figure 19, Zn deposition on ZA@3D-nanoCu substrates forms a smooth and dense layer at 10 mAh cm^− 2^ compared to the rough surface on Cu foil. This improved morphology results from the interaction between 3D Cu nanowires and zincophilic Sb nanoparticles that promotes more uniform zinc deposition. Based on Figure 19b, the core level electron of Zn 2p_1/2_ and Zn 2p_3/2_ shifted towards higher binding energy that indicates a strong interaction between Zn and Sb. Indeed, the shifting is attributed to the effect of the more electronegative Sb [128]. Similarly, Hong et al. modified the zinc plate anode by incorporating a thin layer of metallic Sb as shown in Figure 20 [129]. This protective coating consisted of stacked Sb nanoparticles that acted as zincophilic seeds. They found that the wettability was significantly enhanced, reducing the contact angle from 98.1° to 58.0°. Additionally, the Zn@Sb anode exhibited high surface free energy which facilitated better interaction with zinc ions. Consequently, the nucleation overpotential was measured at only 39.6 mV compared to 142.2 mV for the bare Zn anode. This substantial reduction indicates a lower nucleation energy barrier. For carbon-based materials such as CNTs, rGO and carbon fiber, although they offer high surface area, they have poor zincophilic properties due to large energy barrier of zinc nucleation on carbon substrates. Recently, carbon-based hybrid fiber (Cu@HLCF) containing zincophilic Cu nanoparticles has been developed by Zhou et al. [130]. In this modification, the Cu nanoparticles act as zincophilic seeds for uniform zinc nucleation.

Ultimately, it is important to optimize the surface characteristics through efficient zincophilic modifications. Achieving predominance of zincophilic sites over zincophobic can further improve the stability and reversibility of the zinc anode. However, current research on the mechanisms and key factors driving zincophilicity and zincophobicity remains limited which leaves the gaps in understanding. Moreover, the stability of these zincophilic sites is a concern as they may degrade over time due to chemical reactions with the electrolyte or changes in the electric field during cycling. To ensure long-lasting ZABs performance, it is critical to enhance the long-term durability of these surface modifications.

## Recommendation

Currently, experimental test procedures and parameters for zinc anodes lack standardization which leads to inconsistencies performance results across various studies. For example, variations in electrolyte volume, areal capacity and current density during galvanostatic charge-discharge impact the outcomes of both symmetric and full-cell evaluations. Increasing the electrolyte-to-anode ratio in symmetric cell enhanced cycling stability and reduced dendrite formation [131]. Admittedly, increasing electrolyte content help maintain stable solid electrolyte interface layer, which is essential for long-term cycling stability [132]. However, it is failed to align well with the conditions of real-world applications where electrolyte usage is often constrained. Modifying the areal capacities during charge-discharge cycles could lead to highly variable capacity retention measurements. These inconsistencies highlight the urgent need for standardized testing method to facilitate cross-study comparisons to ensures fair benchmarking for practical use of ZABs.

The primary metrics to evaluate zinc anodes is capacity retention which directly indicates how well the battery can maintain performance over repeated charge-discharge cycles. However, this parameter is often neglected or inconsistently reported. Capacity retention is crucial to assess the long-term effectiveness of zinc anodes, particularly for applications in ZABs where reversible cycle is critical. Without uniformity in testing conditions such as current density and cycle condition, the reported values for capacity retention lack reproducibility, making it difficult to assess the relative performance of different anode designs. For example, capacity retention of 85% after 100 cycles is achievable at this low current density. However, when current density is increased to 5 mA cm^−2^, the capacity retention drops significantly, often to around 60% [133,134].

Furthermore, key operating parameters such as temperature and depth of discharge are frequently overlooked in experimental designs, despite their significant impact on zinc anode performance. ZABs operate under fluctuating conditions in open system. Optimizing these parameters ensure reliable performance in diverse environments, from low-temperature climates to high-heat industrial applications. Operating temperatures above certain threshold accelerate side reactions such as hydrogen evolution and zinc corrosion, leading to rapid degradation of the zinc anode. However, Zhao et al. [135] noted that ZABs demonstrate cycling feasibility at 80°C. Likewise, Liu et al. [136] found Zn||Zn symmetric cells demonstrated remarkable stability, sustaining over 3642 h at room temperature and over 112 h at 80°C. At the same time, deeper discharge cycles increase the likelihood of dendrite formation and capacity fading. Under harsh conditions of 92% depth of discharge, zinc battery cells exhibit stable cycling but are prone to dendrite formation, which can be mitigated by specific electrolyte additives [137]. Higher charge rates also tend to cause more dendritic growth [138]. The absence of standardized guidelines for these parameters contributes to the variability in reported results and limits the broader applicability.

In terms of cell design and configuration, most studies rely heavily on button cells to evaluate the cycling performance of zinc anodes in ZABs. While button cells offer simplicity and ease of assembly, they fail to accurately represent the behaviour of larger and more complex battery systems. The small size of button cells results in distinct thermal and mechanical properties compared to cylindrical, prismatic and pouch cells, which are commonly used in commercial applications. For example, cylindrical cells may experience uneven current distribution due to their geometric configuration [139], while pouch cells have large surface area in direct contact with neighbouring cells, which can lead to strong heat transfer [140]. These differences can influence the performance and degradation mechanisms of zinc anodes, highlighting the need for testing procedures that incorporate larger-scale cell designs.

To address these challenges, concerted effort is required to harmonize experimental procedures and establish standardized parameters for zinc anode testing. This includes defining electrolyte compositions, operating temperatures and cycling condition, as well as ensuring consistent reporting of key metrics like capacity retention, Coulombic efficiency and cycle life. Advanced characterization techniques can provide real-time insights into the chemical and structural evolution of zinc anodes during cycling, enabling researchers to identify degradation mechanisms and optimize anode designs more effectively. In addition, extending studies beyond button cells to include larger-scale configurations such as cylindrical, prismatic and pouch cells will provide more realistic assessment of zinc anode performance under practical conditions. This approach not only bridges the gap between laboratory-scale research and commercial applications but also facilitates the identification of scale-dependent challenges such as thermal management and mechanical stability.

## Conclusion

ZABs face significant challenges due to the limitations of bare zinc anodes. While ZABs is advantageous for its low cost and high volumetric energy density, it suffers from persistent issues related to zinc anode such as uneven zinc deposition, dendrite growth and hydrogen evolution reactions. This review provides a detailed explanation of above issues and delves into the underlying mechanisms. For this reason, various strategies for improving zinc anodes have been explored. Recent study has investigated methods such as alloying anode, nanoporous design and zincophilic design. This review comprehensively summarizes above approaches, discussing the respective advantages, drawbacks and offering recommendations for future improvements. Despite that, research on zinc anodes on ZAB system is still in the early stages. Many aspects of modification strategy remain underexplored. Therefore, there is an urgent need for continued research to address the gaps in current knowledge and develop more effective solutions. Advancing this field requires a concerted effort to explore new materials, refine modification techniques and improve practical implementation. The potential for combining multiple strategies to enhance zinc anode performance offers promising avenues for future research. While individual modifications have shown significant improvements in electrochemical behaviour, their synergistic effects hold the potential for even greater advancements. By combining these approaches, it is possible to leverage their complementary benefits. For example, integrating porous structures with zincophilic surfaces can improve ionic flux and ensure uniform zinc deposition, while alloying can further enhance cycle stability. A key study by Meng et al. explored the development of eutectic Al_82_Cu_18_ (at%) alloy to fabricate porous structure by dealloying method. This porous architecture not only enhanced electron transfer kinetics but also leveraged the alloying effect to form localized galvanic couples between Cu and intermetallic compounds, effectively mitigating uneven zinc plating. Similarly, surface alloying effects during electrochemical cycling were shown to produce zincophilic sites, which guided reversible and dendrite-free Zn stripping and plating. These combined modifications highlight the potential of synergistic strategies to enhance the performance of zinc anodes that can be applied for ZABs.

However, integrating multiple strategies requires precise optimization of fabrication processes which introduces complexity and potentially increases production costs. Ensuring compatibility between alloying components, porous structures and zincophilic surfaces is critical, as mismatched properties can induce instability. The addition of foreign elements may reduce the availability of zinc ions, limiting their role as active species during electrochemical cycling of ZABs. Although porous surface with abundant zincophilic sites provides high active area to effectively suppress dendrite formation, it often exhibits elevated chemical activity, leading to severe side reactions over prolonged cycling. Additionally, excessive incorporation of alloying elements or zincophilic materials can diminish capacity, as these components do not actively participate in redox reactions. While each modification delivers distinct benefits, their combined effects may not always yield additive improvements and overlapping functionalities can sometimes result in diminishing returns. Achieving optimal balance among alloying, porosity and zincophilic properties requires extensive research and experimental validation, potentially prolonging development timelines.
